# Improve health care access is possible. A case report

**DOI:** 10.1192/j.eurpsy.2021.2092

**Published:** 2021-08-13

**Authors:** O. Gori, R. Raggini, R. Sant’Angelo, G. Tricomi, G. De Paoli, S. Grittani, G. Piraccini

**Affiliations:** 1 Mental Health Department, AUSL ROMAGNA, Cesena, Italy; 2 Uo Npi Cesena, Ausl Romagna, Cesena, Italy; 3 Uo Npi Rimini, Ausl Romagna, Rimini, Italy

**Keywords:** Video modeling, autism spectrum disorder, intellectual disability, access to care

## Abstract

**Introduction:**

Within the hub and spoke organizational model provided by the Emilia Romagna Region for assistance to people with autism spectrum disorder (ASD), Cesena ward of psychiatry represents the hospital Hub. Here, a dedicated team (doctors, psychologist, case manager) creates individualized pathways to ensure second-level specialist diagnostics and the management of comorbidities affecting subjects diagnosed with Autism Spectrum Disorder and Intellectual Disability (ID).

**Objectives:**

We report the case of a 23-year-old man, who from the age of 6 was opposed to any instrumental diagnostic investigation.

**Methods:**

In order to guarantee the patient’s full collaboration in carrying out essential diagnostic activities, short behavioural paths were created including video modelling. The Vi.Co app was used and a new app was created to target behaviors that could not be included in Vi.Co

**Results:**

It was thus possible to make the patient compliant with the execution of blood samples, ECG, MRI of the brain in sedation and CT dental scan.

**Conclusions:**

In our case, communication support systems and behavioral strategies have proved to be excellent allies in significantly improving the quality of care for our young patient. Considering the worst prognosis of pathologies and the reduced life expectancy of subjects suffering from ASD and ID, known in the literature, in our opinion, the first essential step becomes facilitating access to care for these patients.

**Disclosure:**

No significant relationships.

